# Medical Mistrust, COVID-19 Stress, and Intent to Vaccinate in Racial–Ethnic Minorities

**DOI:** 10.3390/bs12060186

**Published:** 2022-06-10

**Authors:** Charlene Minaya, Dean McKay, Hannah Benton, Judite Blanc, Azizi A. Seixas

**Affiliations:** 1 Department of Psychology, Fordham University, Bronx, New York, NY 10458, USA; mckay@fordham.edu (D.M.); hbenton5397@gmail.com (H.B.); 2 Department of Psychiatry and Behavioral Sciences, University of Miami Miller School of Medicine, Miami, FL 33136, USA; juditeblanc@miami.edu

**Keywords:** medical mistrust, vaccine hesitancy, COVID-19, racial/ethnic minorities

## Abstract

Members of the Black, Asian, and Latinx community have been particularly vulnerable to the COVID-19 pandemic but may be hesitant to vaccinate. In a December 2020 study in Black, Asian, and Latinx adults in the U.S. (*n* = 779), only 50% of Black respondents endorsed intending to vaccinate against COVID-19, followed by 65% and 75% of Latinx and Asian participants, respectively. Medical mistrust, fears about COVID-19 contamination, and a proclivity for compulsive checking behaviors related to COVID-19 were significant predictors of intent to vaccinate in Black respondents. Similarly, Asian respondents’ intent to vaccinate was predicted by medical mistrust, fears of the dangerous nature of the virus, and xenophobic concerns about viral spread. In Latinx participants, medical mistrust and compulsive checking for COVID-19-related information were significant predictors of intent to vaccinate. Our findings identify specific behaviors, attitudes, and beliefs we can target to inform community-wide outreach and increase the uptake of COVID-19 vaccines.

## 1. Introduction

Coronavirus disease 2019 (COVID-19) has led to a global public health crisis with widespread health and economic consequences [1,2]. Disproportionate rates of transmission and the lethality of COVID-19 in individuals of racial–ethnic minority groups, particularly African Americans, Asian Americans, and Hispanic/Latinx individuals, suggest that COVID-19-related stress (COVID-19 stress) may be especially prominent for these groups [3]. This disproportionate impact has also highlighted critical, pre-existing racial disparities in the United States. Members of these groups often live in high-density areas and occupy essential worker roles as they are less likely to have advanced educational degrees or higher levels of income, posing additional risks to their health [4]. Furthermore, individuals of racial–ethnic minority groups often have a higher burden of preexisting medical conditions (e.g., obesity, hypertension, diabetes, and respiratory disease) [5,6,7,8] that increase their risk of severe COVID-19 disease course and mortality compared with non-Hispanic Whites.

Despite their elevated COVID-19 infection and mortality risk, certain racial–ethnic minority groups are more likely to engage in vaccine hesitancy or refusal relative to non-Hispanic Whites [9]. While an estimated 83% of eligible Black adults, 84% of eligible Latinx adults, and 97% of eligible Asian adults in the U.S. were fully vaccinated as of March 2022 (most recent survey date), only 70% of Asian, 45% of Black, and 43% of Latinx individuals aged 12 years and older had received a first booster dose of the COVID-19 vaccine as of May 2022 [10]. Preliminary evidence indicates that medical mistrust is an important component of vaccine hesitancy [9]. Although minority health disparities have often been attributed to the financial burden of medical care in the U.S. and limited access to such care, the historical mistreatment of racial minorities and resulting medical mistrust (i.e., the belief that members of the healthcare system and its agent are untrustworthy and harmful) also play an important part in reluctant engagement with the medical system [11]. Given the long-standing history of discrimination against racial–ethnic minorities in the United States, mistrust in healthcare is hardly unwarranted [12]. Early nonconsensual research on enslaved people in the U.S. formed the basis for certain practices in medical subdisciplines such as gynecology and extended into the 20th century with infamous examples of unethical medical and research misconduct (e.g., Tuskegee syphilis experiment [13] and the U.S. study infecting Guatemalans with a sexually transmitted disease [14]), contributing to mistrust in the healthcare system. As these groups are also more likely to contract and die from COVID-19 [15], research into COVID-19 vaccine hesitancy in these groups is critical.

While historical mistreatment of racial–ethnic minority groups in healthcare may have contributed to medical mistrust, lower participation in experimental or clinical trials, and lower healthcare utilization behaviors [16], the key drivers of vaccine hesitancy remain unclear [17]. To our knowledge, there is a dearth of evidence explaining individual-level and group-based differences in COVID-19 vaccine uptake among racial–ethnic minorities. The lack of a clear and systematic mechanism explaining the causes of vaccine hesitancy has grave consequences and prevents the identification of critical targets to overcome this hesitancy. The purpose of this study, therefore, is to investigate psychological (COVID-19 stress: risk perception beliefs, fears, socioeconomic stress, and trauma) and attitudinal (medical mistrust) drivers of vaccine hesitancy among Black/African Americans, Asian Americans, and Hispanic/Latinx individuals. Identifying group-specific drivers of vaccine hesitancy targets provides clear intervention targets to ameliorate COVID-19 vaccine uptake among these groups.

## 2. Methods

### 2.1. Participants

Black/African American (hereafter referred to as Black), Asian American (hereafter referred to as Asian), and Hispanic/Latinx (hereafter referred to as Latinx) participants were recruited from the 12th of November to 14 December 2020, via Qualtrics, an online survey platform, as part of the Insights on how COVID-19 Affects Racial Ethnic (i-CARE) (for) minorities’ mental health study, approved by the NYU Grossman School of Medicine IRB. The sample reflects a wide distribution of zip codes across the United States. Participants received USD 6 compensation from panel providers upon completion of the survey. Study eligibility criteria included: being 18 years of age or older, proficiency in English or Spanish (survey available in both languages), identifying primarily as Black, Asian, or Latinx, and providing informed consent.

Participants who failed to meet inclusion criteria were excluded from the study. Of the 806 respondents, 27 were eliminated based on failure to complete at least 80% of the survey and careless or automated responding (e.g., reCAPTCHA score lower than 0.5, the default threshold). The reCAPTCHA score ranges from 0.0 to 1.0 and is utilized to distinguish between human users and bots [18]. Higher scores indicate a greater likelihood that the user is human and scores greater than 0.5 suggest a human user.

### 2.2. Measures

Demographic questions. Participants were asked to provide assorted demographic information including age, gender, highest educational degree, and political party affiliation.

COVID-19-related questions. Participants were asked COVID-19-related questions around news exposure, whether they personally contracted COVID-19 or knew anyone who had contracted or died from the disease, and adherence to CDC guidelines around physical distancing and adequate hygiene. Intention to vaccinate against COVID-19 was inquired with the following yes/no item: (i) If a free or affordable COVID-19 vaccine deemed safe and effective becomes available, will you get vaccinated?

Group-Based Medical Mistrust Scale (GBMMS) [19]. The GBMMS contains 12 items and assesses ethnic-based mistrust in healthcare systems (i.e., mistrust of medical professionals, unwillingness to seek medical treatment, and healthcare dissatisfaction). Respondents rated items on a 5-point Likert scale (1 to 5), four items were reverse coded, and each item was summed for a global mistrust score. Higher scores on this measure denote higher levels of ethnic-based medical mistrust. The GBMMS has demonstrated psychometric soundness in studies involving Latinx [19] and Black [20] populations but has not been widely studied in Asian groups. In this study, the GBMMS demonstrated convergent validity with the MMI and good reliability (Cronbach’s α = 0.86) in the overall sample and in the Black (α = 0.81), Asian (α = 0.89), and Latinx (α = 0.86) groups.

Medical Mistrust Index (MMI) [21]. The MMI is a 15-item measure that assesses general mistrust in healthcare systems. Participants rated statements on a 5-point Likert scale with six reverse-coded items. The rating system was revised from the original 4-point scale to a 5-point scale to include a “Neutral” option. Item ratings were summed for an overall mistrust score with higher scores indicating greater mistrust. The MMI has demonstrated adequate reliability and validity in studies of Black [21,22] and Latinx [23] populations. However, it has not been extensively investigated in Asian groups. In this study, the MMI demonstrated good reliability (α = 0.85) in the total sample and in each group (Black: α = 0.79; Asian: α = 0.87; Latinx: α = 0.86).

COVID-19 Stress Scales (CSS) [24]. The CSS assesses COVID-19-related fears and distress using 36 items that yield a five-factor structure: (i) fear of danger and contamination, (ii) worry about socioeconomic costs, (iii) xenophobic fears, (iv) traumatic stress, and (v) compulsive checking. There is no factor analytically derived total score. Respondents were instructed to endorse the presence of COVID-19-related fears or worries within the past 7 days. Items were rated on a 5-point Likert ranging from 0 to 4. Items were summed to yield 1 global score and 5 subscale scores (related to the five-factor structure), and higher scores indicated greater COVID-19-related stress. The CSS demonstrated good reliability and validity in development and validation, which included Black, Asian, and Latinx individuals from the United States. In this study, the CSS demonstrated excellent reliability in the overall sample and within each group (all subscale αs > 0.92).

### 2.3. Data Analysis

We hypothesized that the three groups would significantly differ on individual-level (MMI) and group-based medical mistrust (GBBMS), COVID-19 stress (CSS), and intent to vaccinate against COVID-19. We also hypothesized that greater medical mistrust (individual: MMI and group: GBMMS) would negatively predict intent to vaccinate, and higher COVID-19 stress (CSS) would positively predict intent to vaccinate in each group. Lastly, we hypothesized that the predictors of vaccine hesitancy would differ across the three groups due to aforementioned cultural and historical differences in experiences with the healthcare and medical community.

In order to test these hypotheses, we conducted hierarchical and logistic regression analyses, analysis of variance (ANOVA), and chi-square tests; where relevant, statistical tests were two-tailed. As age, gender, educational degree, and political party affiliation have been related to vaccine hesitancy in prior studies [25], these variables were treated as covariates in the aforementioned analyses when significantly associated with vaccine hesitancy in this study (see “Covariates” subsection in results section). Significant findings for ANOVAs conducted were probed with the Tukey HSD test [26], as there are no predicted specific ethnic group differences for the assessments related to medical mistrust, vaccine hesitancy, or COVID-19 stress.

## 3. Results

The final sample consisted of 779 participants, the majority of whom were female (406) (52.1%). The mean age of respondents was 39.52 years (*n* = 775; *SD* = 14.54) and ranged from 18–91 years. Racial–ethnic composition of the sample was diverse, with 270 (34.7%) respondents identifying as Black, 262 (33.6%) as Asian, and 247 (31.7%) as Latinx. The majority of Black respondents were female (62.6%) and reported a political affiliation with the Democratic party (70.7%). Almost half (45.2%) of Black respondents reported attaining at least a high school degree. Asian respondents were primarily male (66.4%) with 47.3% belonging to the Democratic party and 42% possessing a four-year college degree (as their highest degree). The majority of Latinx respondents were female (60.3%) and almost half (49%) of Latinx respondents reported an affiliation with the Democratic party. Similarly to Black respondents, more Latinx subjects reported that their highest educational degree attained was a high school degree (44.5%). Refer to Table 1 for demographic breakdowns for each group. Descriptive and inferential statistics were conducted using IBM SPSS version 25.0. Refer to Table 2 for descriptive statistics for each group.

### 3.1. Covariates

Chi-square analyses were conducted to compare the three groups on gender, academic degrees, and political party affiliation. A one-way ANOVA and Tukey’s HSD were utilized to compare group differences related to age. There were significant group differences on age (*F*(2, 772) = 14.40, *p* < 0.001), gender (*X*^2^(2, *n* = 779) = 54.59, *p* < 0.001), highest educational degree attained (*X*^2^(10, *n* = 779) = 87.68, *p* < 0.001), and political party affiliation (*X*^2^(6, *n* = 779) = 39.52, *p* < 0.001). Asian respondents were significantly older, possessed more advanced educational degrees, and included more males compared with both Black and Latinx groups (see Table 1). Black individuals were significantly more likely to self-identify as Democratic than Asian and Latinx participants (see Table 1).

Age, gender, and education (but not political affiliation) were significantly related to intent to vaccinate for Latinx individuals. However, age, gender, education, and political party were not significantly related to intent to vaccinate for Black or Asian respondents. Subsequent analyses controlled for age, gender, and education for Latinx participants by including them as covariates.

### 3.2. Group Differences on Medical Mistrust

**Figure 1:**
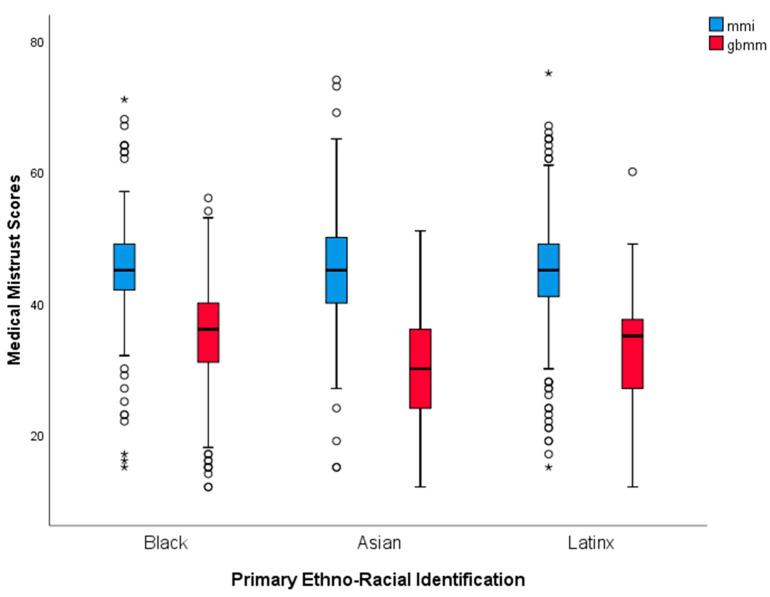
This figure depicts Black, Asian, and Latinx participants’ medical mistrust scores on the Medical Mistrust Index and the group-based medical mistrust scale. Each asterisk represents an outlier identified in the data. * extreme outliers.

Black, Latinx, and Asian groups did not significantly differ from each other on individual-level medical mistrust (MMI), *F*(2, 778) = 0.44, *p* = 0.647. All three groups significantly differed from each other on group-based medical mistrust (GBMMS), *F*(2, 778) = 29.51, *p* < 0.001, as GBMMS scores were significantly related to racial–ethnic groups. Asian respondents scored significantly lower on group-based medical mistrust than Latinx and Black participants, while Black respondents scored significantly higher than the other two groups. Refer to Figure 1 or Table 2 for each group’s medical mistrust averages.

### 3.3. Group Differences on COVID-19 Stress

Analyses of variance indicated that there were significant group differences in COVID-19 stress components. To investigate these COVID-19 stress-related differences, we conducted post hoc analyses using Tukey’s honestly significant difference (HSD) tests.

*COVID-19-related danger and contamination.* On this subscale, Asian respondents reported the lowest levels, followed by Black and Latinx participants (refer to Table 2). Latinx individuals reported significantly higher danger and contamination-related worries relative to Asian participants (mean difference = 3.59, *p* = 0.003; *F*(2, 778) = 5.48, *p* = 0.004). Black subjects, however, did not significantly differ from either Asian (*p* = 0.411) or Latinx (*p* = 0.100) groups on their levels of danger and contamination concerns.

*Socioeconomic concerns.* The groups significantly differed on this subscale, *F*(2, 778) = 24.63, *p* < 0.001. Asian respondents averaged the lowest socioeconomic concerns, followed by Black and then Latinx subjects (see Table 2). Asian individuals scored significantly lower on socioeconomic concerns than Black (mean difference = −3.47, *p* < 0.001) or Latinx (mean difference = −4.17, *p* < 0.001) participants.

*Xenophobic fears.* There were significant group differences regarding COVID-19-related xenophobic fears, *F*(2, 778) = 5.35, *p* = 0.005. Asian subjects scored the lowest on xenophobic fears, followed by Latinx and then Black participants (refer to Table 2). Asian individuals scored significantly lower than Latinx (mean difference = −1.63, *p* = 0.030) and Black (mean difference = −1.91, *p* = 0.007) respondents, but no other groups significantly differed from each other.

*Traumatic stress.* There were significant group differences regarding COVID-19-related traumatic stress, *F*(2, 778) = 5.86, *p* = 0.003. Asian participants scored the lowest on this subscale, followed by Latinx and then Black respondents (see Table 2). Asians demonstrated significantly lower levels of traumatic stress compared with Latinx (mean difference = −1.55, *p* = 0.026) and Black (mean difference = −1.89, *p* = 0.004) counterparts.

*Compulsive checking.* The three groups significantly differed on this subscale, *F*(2, 778) = 9.29, *p* < 0.001. Asians averaged lower scores on compulsive checking, followed by Latinx and Black participants (see Table 2). While there were no significant differences between Black and Latinx participants, Asians scored significantly lower than Latinx (mean difference = −1.73, *p* = 0.008) and Black (mean difference = −2.35, *p* < 0.001) participants on compulsive checking.

**Figure 2:**
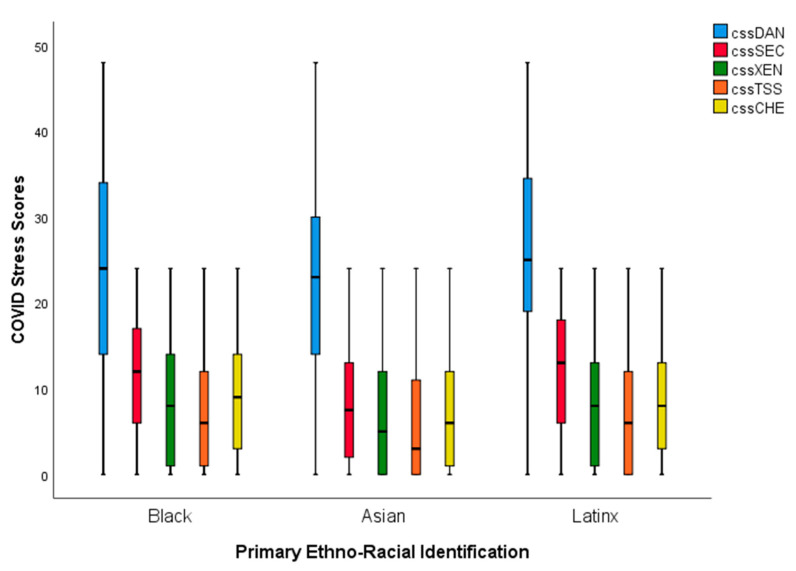
This figure depicts Black, Asian, and Latinx participants’ COVID-19 stress scores on the COVID-19 Stress Scales.

On all CSS subscales, Asian participants demonstrated significantly lower levels of COVID-19 stress, followed by Black or Latinx participants. Black and Latinx participants did not significantly differ from each other on worries related to socioeconomic consequences during the pandemic (*p* = 0.514), xenophobic fears (*p* = 0.902), traumatic stress (*p* = 0.837), and compulsive checking (*p* = 0.522). In relation to subscales related to worries about danger and contamination and socioeconomic consequences, Latinx participants reported higher levels than Black respondents while this pattern was reversed for xenophobic fears, traumatic stress, and compulsive checking (although these differences were not significant, See Figure 2).

## 4. Group Differences in Intent to Vaccinate

**Figure 3:**
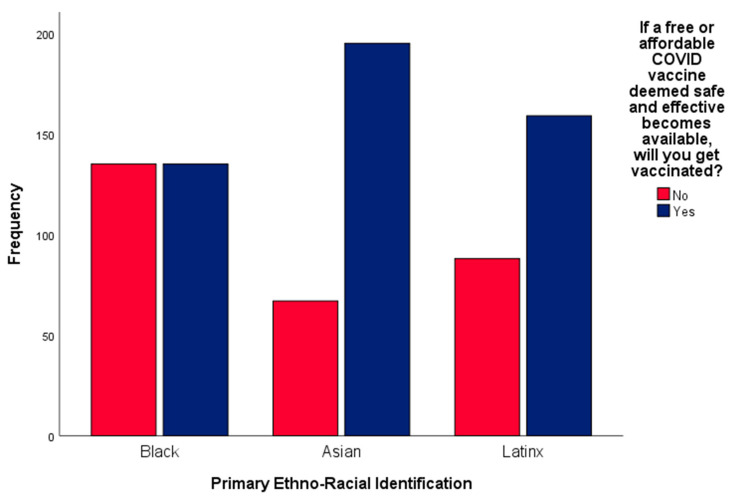
This figure depicts Black, Asian, and Latinx participants’ reported intent to vaccinate against COVID-19.

We conducted a chi-square test of independence to investigate the relationship between racial–ethnic minority status and intent to vaccinate. Racial–ethnic group membership was significantly related to intent to vaccinate, *X^2^*(2, *n* = 779) = 34.35, *p* < 0.001. Asians were significantly more likely to endorse vaccination (195 (74.4%)) compared with both Black (135 (50%)) and Latinx (159 (64.4%)) groups. Black respondents were significantly less likely to endorse intent to vaccinate (135 (50%)) than both Asian (67 (25.6%)) and Latinx (88 (35.6%)) participants (See Figure 3).

### 4.1. The Effect of Medical Mistrust on Intent to Vaccinate

Logistic regression analysis for each group was conducted to investigate the relationship between medical mistrust and intent to vaccinate. Individual-level medical mistrust (MMI) was significantly negatively associated with intent to vaccinate in each group: Black (*X*^2^(1, *n* = 270) = 4.61, *p* = 0.032; *B* = −0.03, OR = 0.97, *p* = 0.036), Asian (*X*^2^(1, *n* = 262) = 6.46, *p* = 0.011; *B* = −0.04, OR = 0.96, *p* = 0.013), and Latinx (*X*^2^(4, *n* = 245) = 29.26, *p* < 0.001; *B* = −0.06, OR = 0.94, *p* < 0.001). Our results indicate that individuals with greater individual-level medical mistrust had lower intent to accept a COVID-19 vaccine, with Blacks with the lowest odds (13%) reduction and Latinx (16%) with the highest odds reduction.

Group-based medical mistrust (GBMMS) was also significantly negatively associated with intent to vaccinate in all three groups: Black (*X*^2^(1, *n* = 270) = 5.93, *p = 0*.015; *B* = −0.04, OR = 0.96, *p* = 0.017), Asian (*X*^2^(1, *n* = 262) = 14.27, *p* < 0.001; *B* = −0.07, OR = 0.94, *p* < 0.001), and Latinx (*X*^2^(4, *n* = 245) = 19.45, *p* = 0.001; *B* = −0.04, OR = 0.97, *p* = 0.033). These findings indicate that both individual-based medical mistrust and group-based medical mistrust demonstrate significant relationships with intent to vaccinate in each group.

### 4.2. The Effect of COVID-19 Stress on Intent to Vaccinate

Multiple logistic regression models with the five COVID-19 stress factors as predictors indicated that specific factors were significantly related to intent to vaccinate in Black (*X*^2^(5, *n* = 270) = 28.72, *p* < 0.001), Asian (*X*^2^(5, *n* = 262) = 35.07, *p* < 0.001), and Latinx (*X^2^*(8, *n* = 245) = 32.17, *p* < 0.001) groups. COVID-19-related socioeconomic concerns (Black: *p* = 0.561, Asian: *p* = 0.495, Latinx: *p* = 0.221) and traumatic stress (Black: *p* = 0.204, Asian: *p* = 0.558, Latinx: *p* = 0.355) did not significantly predict intent to vaccinate in any of the three groups.

Worries about danger and contamination were positively associated with intent to vaccinate for both Black (*B* = 0.04, OR = 1.04, *p* = 0.031) and Asian (*B* = 0.10, OR = 1.10, *p* < 0.001) respondents but not for Latinx respondents (*p* = 0.060). Compulsive checking was positively related with intent to vaccinate in Black (*B* = 0.11, OR = 1.11, *p* < 0.001) and Latinx respondents (*B* = 0.09, OR = 1.10, *p* = 0.013) but not Asian respondents (*p* = 0.496). Meanwhile, xenophobic fears were negatively associated with intent to vaccinate among Asian respondents alone (*B* = −0.17, OR = 0.85, *p* < 0.001; Black: *p* = 0.101, Latinx: *p* = 0.550).

The combined effect of Medical Mistrust and COVID-19 Stress on Intent to Vaccinate among Black Participants

In our analysis to investigate the combined effect of medical mistrust and COVID-19 stress across the three racial–ethnic groups, we found that worries about danger and contamination (*B* = 0.01, *p* = 0.23), compulsive checking (*B* = 0.07, OR = 1.07, *p* = 0.003) and individual-level medical mistrust (MMI) (*B* = −0.04, OR = 0.97, *p* = 0.038) predicted intent to vaccinate among Blacks (*X^2^*(3, *n* = 270) = 25.20, *p* < 0.001). While in another model, group-based medical mistrust (GBMMS) (*B* = −0.05, OR = 0.96, *p* = 0.008), danger and contamination concerns (*B* = 0.01, *p* = 0.26), and compulsive checking (*B* = 0.07, OR = 1.03, *p* = 0.001) predicted intent to vaccinate (*X^2^*(3, *n* = 270) = 27.99, *p* < 0.001). Danger and contamination concerns were no longer significant in either model, but compulsive checking and medical mistrust remained significant.

### 4.3. Medical Mistrust and COVID-19 Stress on Intent to Vaccinate among Asian Participants

In another race–ethnicity stratified analysis with Asian participants, we found that danger and contamination concerns (*B* = 0.10, OR = 1.10, *p* < 0.001), xenophobic fears (*B* = −0.15, OR = 0.86, *p* < 0.001), and individual-level medical mistrust (MMI) (*B* = −0.05, OR = 0.95, *p* = 0.013) predicted intent to vaccinate against COVID-19 (*X^2^*(3, *n* = 262) = 39.55, *p* < 0.001). While in another multiple regression model, worries about danger and contamination (*B* = 0.10, OR = 1.10, *p* < 0.001), xenophobic fears (*B* = −0.11, OR = 0.89, *p* = 0.002), and group-based medical mistrust (GBMMS) (*B* = −0.07, OR = 0.93, *p* = 0.003) predicted intent to vaccinate in Asian individuals (*X^2^*(3, *n* = 262) = 42.65, *p* < 0.001).

### 4.4. Medical Mistrust and COVID-19 Stress on Intent to Vaccinate among Latinx Participants

Among Latinx, compulsive checking (*B* = 0.10, OR = 1.11, *p* < 0.001) and individual-level medical mistrust (MMI) (*B* = −0.08, OR = 0.93, *p* < 0.001) predicted intent to vaccinate (*X^2^*(5, *n* = 245) = 46.57, *p* < 0.001). While in another model, group-based medical mistrust (GBMMS) (*B* = −0.07, OR = 0.94, *p* = 0.001) and compulsive checking (*B* = 0.11, OR = 1.12, *p* < 0.001) predicted intent to vaccinate (*X^2^*(5, *n* = 245) = 39.66, *p* < 0.001).

## 5. Discussion

Although medical technology has resulted in multiple viable COVID-19 vaccines at a record pace [27], vaccine hesitancy remains a critical threat to reducing the continued propagation of COVID-19. While technological advances and adoption have facilitated the delivery of health services [28,29], vaccine misinformation, which has been linked to vaccine hesitancy and a decline in immunizations pre-COVID-19 [30], has spread prominently with these advances. According to the WHO, accompanying the pandemic is an ominous and insidious proliferation of misinformation about COVID-19 vaccines, which has spawned an *infodemic*, where individuals are bombarded with misleading information about vaccines leading to confusion, risky health behaviors, and mistrust in healthcare authorities [31]. We believe that the hesitance to be vaccinated among racial–ethnic minorities differs from other groups, especially considering the discrimination members of these groups have historically met with in medical systems. Despite several attempts to dispel misinformation and tailor messaging about vaccines to these groups, vaccine hesitancy rates remain high, raising the possibility that other factors are also responsible for this hesitancy. For example, the perceived rapid development of vaccines (despite following gold-standard procedures during development), acute side-effects of the vaccine, and concerns about the long-term safety of COVID-19 vaccines may be other factors that drive COVID-19 vaccine hesitancy [32]. These concerns highlight important psychological factors (COVID-19 stress) and attitudes (medical mistrust) that contribute to vaccine hesitancy among the general population and specific groups who are more vulnerable to negative COVID-19 outcomes (e.g., racial–ethnic minorities).

Therefore, the purpose of this study was to evaluate the impact of medical mistrust attitudes and COVID-19 stress on vaccine hesitancy in Black, Asian, and Latinx individuals in the U.S. The findings highlight that Black respondents demonstrated the greatest vaccine hesitancy (approximately 50% reporting intent to vaccinate) mirroring results found for other vaccines such as influenza [33], while Asian respondents exhibited the least vaccine hesitancy as approximately three-quarters of these individuals reported an intention to vaccinate. Despite group-based differences in vaccine hesitancy rates, our results highlight other factors that might explain vaccine hesitancy across all three groups. These factors include medical mistrust and COVID-19 stress, a constellation of five psychological factors: (i) fear of danger and contamination, (ii) worry about socioeconomic costs, (iii) xenophobic fears, (iv) traumatic stress, and (v) compulsive checking.

### 5.1. COVID-19 Stress and Vaccine Hesitancy

Our findings indicate that COVID-19 stress is associated with vaccine hesitancy in three racial–ethnic groups (Black, Asian, and Latinx) in the U.S. Among Blacks and Asians, worries about COVID-19-related danger and contamination were positively associated with intent to vaccinate. While among Blacks and Latinx, compulsive checking was positively related with intent to vaccinate. These findings show that there may be unique psychological mechanisms at work, where members of Black and Asian groups with greater fear of contamination and checking behaviors are more likely to accept a vaccine to protect them from COVID-19.

Conversely, COVID-19-related xenophobic fear was negatively associated with vaccine hesitancy in Asians. The impact of xenophobia on vaccine hesitancy in this group may be associated with the fact that this group has been the target of xenophobic-driven hostilities, and the recent spate of xenophobic beliefs and attacks against Asian Americans may increase their vaccine hesitancy [34]. Our findings are particularly insightful because they offer a unique perspective about a potential explanation for low COVID-19 vaccine rates among Asian Americans. It is plausible that lowering the victimization fears of xenophobic beliefs and attacks toward Asians can increase their willingness to receive a COVID-19 vaccine. Coupled with xenophobic fears is the constant worry about danger and contamination. To overcome these unique psychological barriers to vaccine uptake, culturally tailored public health programs and messaging are needed to explicitly address worries and anxiety about contamination and danger and compulsive checking behaviors.

### 5.2. Medical Mistrust and Vaccine Hesitancy

Both medical mistrust measures (group and individual-based) negatively predicted intent to vaccinate in all three groups, suggesting that greater medical mistrust lowers intent to accept the COVID-19 vaccine. Interestingly, while the three groups did not significantly differ in individual-based medical mistrust, differences in group-based medical mistrust (i.e., rendering group identity salient) were observed. Additional research is needed to investigate whether this mistrust may be heightened in medical settings that render this identity salient (such as through a lack of racial–ethnic or linguistic diversity). Black participants had the highest levels of group-based medical mistrust followed by Latinx and Asian individuals. The sordid history of systematic discrimination and prejudice in health settings may be key drivers of group-based medical mistrust, especially for Blacks. Blacks may feel that the development of vaccines via key clinical trials were not inclusive due to underrepresentation of racial–ethnic minorities. While addressing group-based differences in mistrust among Blacks offers a viable solution, the intersection of medical mistrust attitudes and psychological barriers such as COVID-19 stress (worry about danger and contamination) make it harder to craft an appropriate and nuanced program that addresses the complex links between barriers. Solutions cannot be singularly focused but rather diverse in scope, addressing multiple barriers simultaneously.

### 5.3. Limitations

Our findings should be cautiously interpreted in light of several methodological limitations. First, our cross-sectional design prohibited us from capturing longitudinal data and thus precluded us from investigating whether changes in medical mistrust and COVID-19 stress impacted intent to vaccinate over time. Follow-up research with individual interviews and other direct engagement with members of communities historically hesitant regarding vaccinations is warranted. Specifically evaluating the connections between COVID-19 stress and medical mistrust would be useful in refining public health messages to ensure larger proportions of an increasingly diverse U.S. population seeking and receiving the vaccine, particularly considering factors that may impact adoption of misinformation such as age or geographical location. This is especially important in light of the potential that booster vaccines are anticipated [35] to continue to keep COVID-19 and potentially future coronaviruses from escalating to full pandemics.

Another limitation includes the omission of other psychological factors that might impact vaccine hesitancy such as health literacy, discrimination, and implicit medical biases [36]. Our use of educational attainment as a proxy for health literacy is insufficient and it is recommended that future studies assess both general and COVID-19-specific health literacy. Future research should evaluate participants’ personal medical experiences, especially discriminatory experiences, and their relationship to medical mistrust and vaccine hesitancy, as this may provide a critical target in improving relationships between these communities and healthcare providers and promoting everyone’s health and well-being.

## 6. Conclusions

Although COVID-19 factors appear to play a role in acceptance of the COVID-19 vaccine, mistrust in the medical system contributes to vaccine hesitancy. As more lethal and transmissible variants of COVID-19 continue to emerge, vaccine hesitancy poses a major public health threat. Our findings highlight shared and unique psychological drivers and mechanisms of vaccine hesitancy and acceptance in Blacks, Asians, and Latinx and posit the need for community and group-based programs and messages to address historical and on-going injustices in healthcare and concerns about vaccine safety. Our findings add value to a growing body of literature that narrowly focuses on distal and group-based characteristics to address vaccine hesitancy with the unfortunate consequence of ignoring proximal individual-based drivers to vaccine hesitancy.

## Figures and Tables

**Table 1:** This table presents the demographic information for each group.

	Gender (*n* = 270)	Age (*n* = 268)	Education (*n* = 270)	Political Party (*n* = 270)
**Black**	Female: 169 (62.6%) Male: 101 (37.4%)	*M* = 38.07 *SD* = 14.01 Min: 18 Max: 74	High school (equivalent): 122 (45.2%) Two-year college: 55 (20.4%) Four-year college: 62 (23%) Advanced: 27 (10%) Other: 4 (1.5%)	Democratic: 191 (70.7%) Republican: 20 (7.4%) Other: 16 (6%) None: 43 (15.9%)
**Asian**				
	Female: 88 (33.6%) Male: 174 (66.4%)	*M* = 43.33 *SD* = 15.28 Min: 18 Max: 91	High school (equivalent): 46 (17.6%) Two-year college: 36 (13.7%) Four-year college: 110 (42%) Advanced: 64 (24.5%) Other: 6 (2.3%)	Democratic: 124 (47.3%) Republican: 41 (15.6%) Other: 17 (6.5%) None: 80 (30.5%)
**Latinx**				
	Female: 149 (60.3%) Male: 98 (39.7%)	*M* = 37.03 *SD* = 13.47 Min: 18 Max: 81	High school (equivalent): 110 (44.5%) Two-year college: 42 (17%) Four-year college: 60 (24.3%) Advanced: 25 (10.1%) Other: 10 (4%)	Democratic: 121 (49%) Republican: 39 (15.8%) Other: 15 (6%) None: 72 (29.1%)
**Legend**				
**Abbreviation**		**Measure**		
CSSDAN		Danger and Contamination Subscale (COVID-19 Stress Scales)		
CSSSEC		Socioeconomic Concerns Subscale (COVID-19 Stress Scales)		
CSSXEN		Xenophobic Fears Subscale (COVID-19 Stress Scales)		
CSSTSS		Traumatic Stress Subscale (COVID-19 Stress Scales)		
CSSCHE		Compulsive Checking Subscale (COVID-19 Stress Scales)		
GBMMS		Group-Based Medical Mistrust Scale		
MMI		Medical Mistrust Index		

**Table 2:** This table includes the descriptive statistics for the medical mistrust scales, COVID-19 stress scales, and intent to vaccinate for each group.

	Black (*n* = 270)	Asian (*n* = 262)	Latinx (*n* = 247)
MMI	*M* = 45.28 *SD* = 7.81 Min: 15 Max: 71	*M* = 44.71 *SD* = 8.39 Min: 15 Max: 74	*M* = 44.67 *SD* = 9.06 Min: 15 Max: 75
GBMMS	*M* = 35.17 *SD* = 7.89 Min: 12 Max: 56	*M* = 29.67 *SD* = 8.35 Min: 12 Max: 51	*M* = 32.04 *SD* = 8.64 Min: 12 Max: 60
CSSDAN	*M* = 23.72 *SD* = 12.97 Min: 0 Max: 48	*M* = 22.36 *SD* = 11.76 Min: 0 Max: 48	*M* = 25.95 *SD* = 12.16 Min: 0 Max: 48
CSSSEC	*M* = 11.93 *SD* = 7.49 Min: 0 Max: 24	*M* = 8.45 *SD* = 6.65 Min: 0 Max: 24	*M* = 12.63 *SD* = 7.56 Min: 0 Max: 24
CSSXEN	*M* = 8.66 *SD* = 7.51 Min: 0 Max: 24	*M* = 6.75 *SD* = 6.71 Min: 0 Max: 24	*M* = 8.38 *SD* = 7.43 Min: 0 Max: 24
CSSTSS	*M* = 7.64 *SD* = 7.03 Min: 0 Max: 24	*M* = 5.75 *SD* = 6.08 Min: 0 Max: 24	*M* = 7.30 *SD* = 7.10 Min: 0 Max: 24
CSSCHE	*M* = 9.30 *SD* = 6.88 Min: 0 Max: 24	*M* = 6.95 *SD* = 5.99 Min: 0 Max: 24	*M* = 8.68 *SD* = 6.53 Min: 0 Max: 24
Intent to vaccinate	No: 50% (135) Yes: 50% (135)	No: 25.6% (67) Yes: 74.4% (195)	No: 35.6% (88) Yes: 64.4% (159)

## Data Availability

Data involving these findings are available from the corresponding authors upon reasonable request.
